# Birth weight and catch up growth are associated with childhood impulsivity in two independent cohorts

**DOI:** 10.1038/s41598-018-31816-5

**Published:** 2018-09-12

**Authors:** Patrícia P. Silveira, Irina Pokhvisneva, Hélène Gaudreau, Anne Rifkin-Graboi, Birit F. P. Broekman, Meir Steiner, Robert Levitan, Carine Parent, Josie Diorio, Michael J. Meaney

**Affiliations:** 1 Department of Psychiatry, McGill University & Sackler Institute for Epigenetics & Psychobiology at McGill University, Montreal, Quebec Canada; 2 Ludmer Centre for Neuroinformatics and Mental Health, Douglas Mental Health University Institute, McGill University, Montreal, Quebec H4H 1R3 Canada; 3 Singapore Institute for Clinical Sciences, Agency for Science, Technology and Research (A*STAR), Singapore, Singapore; 4 Department of Psychiatry and Behavioural Neurosciences, McMaster University, Hamilton, Ontario L8N 3K7 Canada; 5 Department of Psychiatry, University of Toronto and Centre for Addiction and Mental Health, Toronto, Ontario M5T 1R8 Canada

## Abstract

Individuals born after intrauterine growth restriction (IUGR) are more impulsive towards palatable foods, but it is not clear 1) if IUGR-related impulsivity is specific for foods and solely based on response inhibition and 2) if the development of impulsivity is due to being born IUGR *per se* or to growing up fast in the first few years of life (catch up growth). Children were classified in the IUGR group if the birth weight ratio was below 0.85. Delta z score for BMI was used as a measure of catch up growth. In MAVAN (N = 274), impulsivity was measured by the Information Sampling Task from the Cambridge Neuropsychological Test Automated Battery (IST - CANTAB), and in GUSTO using the Sticker Delay Task (N = 327). There is a significant effect of interaction between being born IUGR and the magnitude of catch up growth on the reflection impulsivity from IST-CANTAB at 60 months, in which greater catch up growth associates with greater impulsivity in the IST fixed condition in IUGR children. The finding was reproduced in children from the GUSTO cohort using the Sticker Delay Task. We confirmed that catch up growth interacts with IUGR, having a major role in the development of impulsivity in the first years of life and influencing inhibitory control and decision making processes.

## Introduction

Poor fetal growth is associated with long term increased risk for several chronic non-transmittable conditions including type II diabetes^1^, metabolic syndrome^2^ and a diversity of mental health problems^3^. We have previously shown that individuals born after intrauterine growth restriction (IUGR) are more impulsive towards a sweet reward at 3 years of age, especially girls^4^. Moreover, impulsivity at 3 years predicts fat intake at 4 years of age^4^. In addition, poor inhibitory control at 18 months of age is associated with food fussiness at 6 years of age^5^. Therefore, impulsivity seems to be an important element influencing feeding behavior^6^ and food choices in IUGR children^7–9^. IUGR is associated with increased intake of non-healthy foods and this behavioral pattern contributes to a progressive deterioration of the metabolic profile in these individuals. However, it is not clear if IUGR-related impulsivity is specific for food and solely based on response inhibition.

As reviewed by Clark and colleagues^10^, impulsivity can be seen as a construct operating at different levels of information processing, including perceptual analysis, goal representation, and response execution^11,12^. Two main paradigms have been predominantly explored in the different behavioral assessment studies: impulsive choice and response inhibition (e.g., the stop signal task)^13,14^. However, the ability to gather and evaluate information before making a decision, known as “reflection impulsivity”^15^, is important for accuracy in a given task and therefore generally affects the precision of the decision-making process independently of the type of choices being made (e.g. food choices, cognitive tests, etc). The less information is sampled before making a decision, the more impulsive is the individual. Reflection impulsivity may mediate the known effects of IUGR on such behaviors^4,16–18^.

When working with fetal growth restriction, a relevant question is if the observed effects are due to the very exposure to paucity of resources *in utero*, or if the period of rapid growth (“catch up growth”) that often follows is involved, or both. Clinical and experimental data suggest that catch up growth is important to define metabolic risk in individuals born IUGR^19–21^. Several studies explored the relationship between being born IUGR and the risk for mental disorders later in life^22–24^. Very little is known though about the effect of catch up growth on these outcomes in the IUGR population born at term. Our hypothesis is that catch up growth interacts with fetal growth, strengthening the association between IUGR and non-food related impulsivity. In this work, our objectives were to investigate 1) if IUGR-related impulsivity is specific for foods and solely based on response inhibition or is also seen in other forms of impulsivity (e.g. reflection impulsivity) and 2) if the development of impulsivity is due to being born IUGR per se or to growing up fast in the first few years of life (catch up growth).

## Methods

### Subjects

We used data from an established prospective birth cohort (Maternal Adversity, Vulnerability and Neurodevelopment - MAVAN)^25^ which followed up the children at different time points in the first years of life in Montreal (Quebec) and Hamilton (Ontario), Canada. Severe maternal chronic illness, placenta previa, and history of incompetent cervix, impending delivery, or a fetus/infant affected by a major anomaly or born at a gestational age less than 37 weeks were exclusion criteria. Approval for the MAVAN project was obtained from obstetricians performing deliveries at the study hospitals and by the ethics committees and university affiliates (McGill University and Université de Montréal, the Royal Victoria Hospital, Jewish General Hospital, Centre hospitalier de l’Université de Montréal, Hôpital Maisonneuve-Rosemont, St Joseph’s Hospital and McMaster University, Hamilton, Ontario, Canada). The study was conducted in accordance with the rules and regulations of the university ethics committees and informed consent was obtained from all participants.

### Procedures

For this study, we used information collected at birth as well as at 60 months of age, and we had complete growth and CANTAB data available for 274 participants. Children came to the laboratory for cognitive testing (CANTAB, see details below). They had their standing height, without shoes, measured (to the nearest 0.1 cm) with the use of a stadiometer (Perspective Enterprises, PE-AIM-101, Portage, Michigan). Body weight, in light clothing, was also measured (to the nearest 0.1 kg) with the use of a digital floor scale (TANITA BF625, Arlington Heights, Illinois). Body mass index (BMI) was calculated as weight in kilograms divided by height in meters squared (kg/m^2^).

### Predictors

Intrauterine growth restriction - The definition of IUGR was based on the birth weight ratio (BWR), namely, the ratio between the observed birth weight and the sex-specific mean birth weight for each gestational age for the local population^26^. A BWR of <0.85 was classified as IUGR^27^.

Catch up growth – We calculated the z-scores for BMI at 60 months according to World Health Organization (WHO) standards^28^. Moreover, z-scores for BMI at birth, adjusted for gestational age, were calculated according to the International Fetal and Newborn Growth Consortium for the 21^st^ Century (INTERGROWTH-21^st^)^29^. The difference (delta) between z-score BMI at 60 months and at birth was used as a measure of catch up growth.

### Outcome

The Information Sampling Task (IST) from the Cambridge Neuropsychological Test Automated Battery (CANTAB) was designed to measure reflection impulsivity and decision making^30^. Rather than relying on speed-accuracy indices, IST measures reflection impulsivity by calculating the probability of the subject selecting the correct answer at the point of decision on the basis of their sampling of information prior to making that decision.

**Figure 1:**
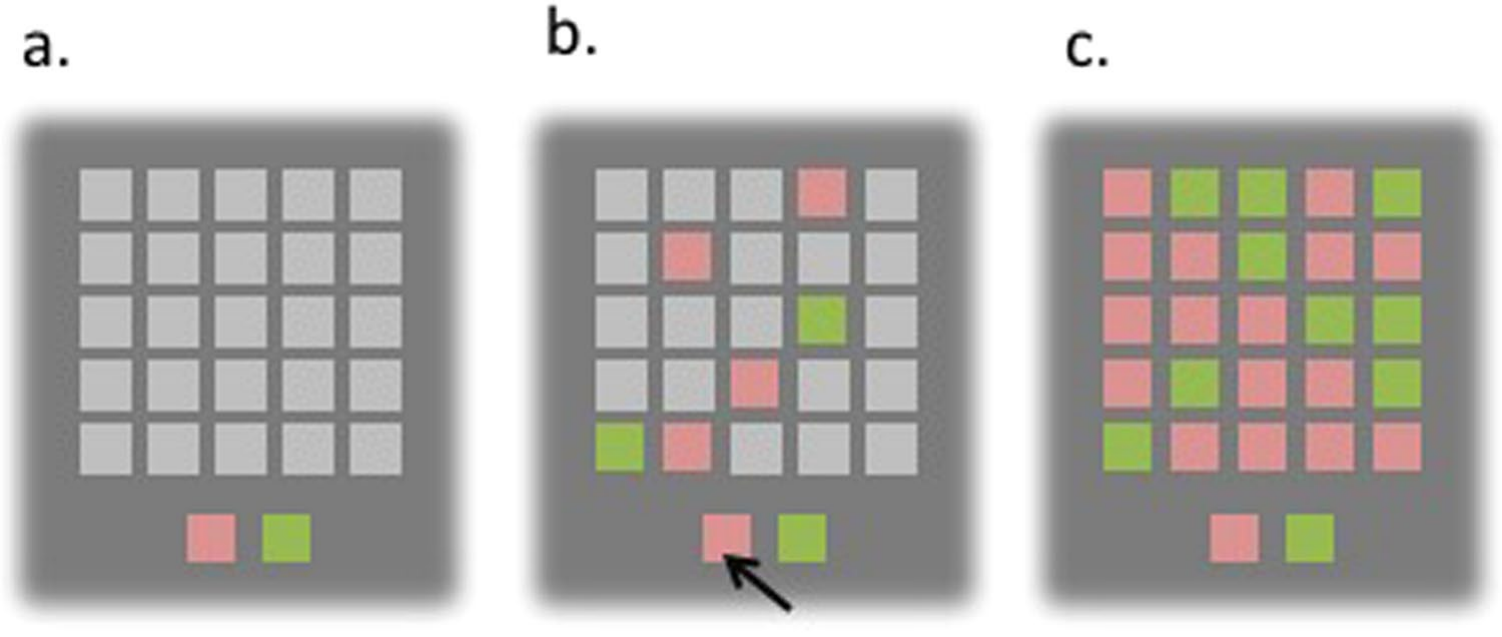
Information Sampling Task. (**a**) Initial screen. The child is instructed to click on the gray boxes to open them, and she can open as many as she wants before deciding which color comprises the majority of the 25 boxes; (**b**) when the child decided that she had enough information to choose which color was more prevalent, she should click on the corresponding color at the bottom; (**c**) all boxes then automatically opened and a message about being correct or incorrect was displayed before the next trial.

On each trial, children are presented with a 5 × 5 matrix of grey boxes on the computer screen, and two larger colored panels at the foot of the screen. The child is told that this is a game for points, won by making a correct decision about which color is in the majority under the gray boxes. Touching a grey box would immediately open that box to reveal one of the two colors displayed at the bottom of the screen. Subjects were able to open boxes at their own rate with no time limit before deciding between one of the two colors, indicating their decision by touching one of the two panels at the bottom of the screen. At this point, the remaining boxes are uncovered and a message is displayed to inform the child whether or not he/she was correct. In the ‘fixed win’ condition, subjects are awarded 100 points for a correct decision regardless of the number of boxes opened. In the ‘decreasing win’ condition, the child loses 10 points for every box opened. In both conditions, they lose 100 points for an incorrect decision. The task starts with a single practice trial, followed by ten trials in each of the two conditions. We were particularly interested in the “fixed” condition for this study, as the children were only 5 years of age by the time of testing and we were interested in their behavior under “non-pressure” or “no gambling” conditions (Fig. 1).

The primary performance outcome measure was the mean probability of being correct at the point of decision (P correct). P correct is the probability that the color chosen by the subject at the point of decision would be correct, based only on the evidence available to the subject at the time (i.e., dependent on the amount of information they had sampled). Recently, the mean P correct formula was updated^31^ and endorsed by the original authors of the measure^32^, therefore in this study we calculated and used the new mean P correct^33^. Other variables analyzed included the mean number of boxes opened per trial, as well as discrimination and sampling errors. Discrimination errors occurred when the participants chose a color that was not at that point in time in the majority, thus making a decision not logically based on the evidence available to them. Sampling errors were the number of trials where the subject chose a color that was not in the overall majority but was in the majority at the point of decision.

### Replication Cohort

#### Subjects

Pregnant women aged 18 years and above were recruited at the National University Hospital (NUH) and KK Women’s and Children’s Hospital (KKH) in Singapore, being of Chinese, Malay or Indian ethnicity with homogeneous parental ethnic background. Mothers receiving chemotherapy, psychotropic drugs or who had type I diabetes mellitus were excluded. The study was conducted according to the rules and regulations of the National University Hospital (NUH) and KK Women’s and Children’s Hospital (KKH) in Singapore ethics committees and approved by these ethics committees. Informed written consent was obtained from each participant. A descriptive paper details other aspects of the cohort^34^. We limited this study to the non-preterm children (born above 37 weeks’ gestation) for the sake of comparison to MAVAN (see MAVAN exclusion criteria above).

#### Procedures

We used information collected at birth as well as at 41 and 48 months of age, and we had complete growth and Sticker Delay data available for 327 participants. Children had their standing height, without shoes, measured (to the nearest 0.1 cm) with the use of a stadiometer, and body weight measured by the research team at 48 months. Body mass index (BMI) was calculated as weight in kilograms divided by height in meters squared (kg/m^2^).

#### Predictors

IUGR and catch up growth were defined and calculated as described in the MAVAN cohort above, but the reference used to calculate BWR in GUSTO was the International Fetal and Newborn Growth Consortium for the 21^st^ Century (INTERGROWTH-21^st^)^29^.

#### Outcome

The average performance on the Sticker Delay Task was adapted from the Snack Delay task administered within Kochanska and colleagues’ battery^35^, and was measured at 41 months of age. On each of the four trials, the child is instructed to place both hands flat on a mat while the experimenter hid a sticker under a clear plastic cup positioned 5 cm away from the top of the mat. The child is told to wait for the experimenter to ring the bell before retrieving the sticker. The average performance is the child’s ability to resist reaching out for the bell or sticker across the different trials.

### Statistical analysis

Statistical analysis of the participants’ baseline characteristics was performed using Student’s T test for continuous data and chi-square tests for categorical variables. Linear regression analyses using IUGR (yes or no) and delta z-score BMI (48 or 60 months – birth) and the interaction term were performed for mean P correct and discrimination errors in MAVAN, and average performance in the Sticker Delay Task in GUSTO. Logistic regressions were run for the presence of sampling errors (yes or no) and number of boxes opened in MAVAN (all boxes opened (24–25) or not). Preliminary analysis adjusted by sex showed no main effect or interaction with sex, therefore for the main analysis boys and girls were analyzed together. In MAVAN, the main outcome measure was the mean P correct variable from the IST during the fixed win condition, but other variables were also similarly analyzed (see description above). No co-variates were used in the regression analysis, as no main differences were seen in the main confounders between the groups (please refer to the Results). Data were analyzed using the Statistical Package for the Social Sciences (SPSS) version 20.0 software (SPSS Inc., Chicago, IL, USA). Significance levels for all measures were set at p < 0.05. The authors will readily share data and protocols with others as requested.

## Results

Children born IUGR or non-IUGR did not differ in baseline characteristics which can be seen in Table 1. The number of boxes opened in the IST was significantly correlated with incorrect judgments in the fixed win condition [r(274) = −0.68, p < 0.0001], demonstrating that response accuracy is a function of the extent of information analysis, a feature of reflection impulsivity.

**Table 1:** MAVAN participants’ characteristics by birth weight group. Data are expressed as means (standard deviations) or number of participants (percentages).

Sample characteristics	Non-IUGRs (n = 229)	IUGRs (n = 45)	p	Correlation or difference BMI z-score at 60 months
Birth weight (kg)^a^	3.51 (0.39)	2.75 (0.24)	<0.0001	r = −366; p < 0.0001
Females (%)^b^	101 (44.1%)	23 (51.1%)	0.39	p = 0.52
Gestational age (weeks)	39.50 (1.19)	39.58 (1.16)	0.70	r = 0.080; p = 0.17
Maternal education – High school or less (%)^b^	104 (46%)	23 (51.1%)	0.62	p = 0.42
Family income below LICO (%)^b^	28 (14.9%)	3 (9.0%)	0.59	p = 0.09
Breastfeeding duration (months)^a^	6.69 (5.15)	5.77 (5.64)	0.40	r = −0.081; p = 0.29
Body mass index z-score at 60 months (kg/m^2^)^a^	0.34 (0.95)	0.24 (1.37)	0.56	1

^a^Student’s t-test and ^b^Chi-square test, or Student’s T-Test when addressing differences in BMI z-score in the different categories. LICO = Low Income Cut Off according to Statistics Canada. Small differences in totals are due to missing information.

**Figure 2:**
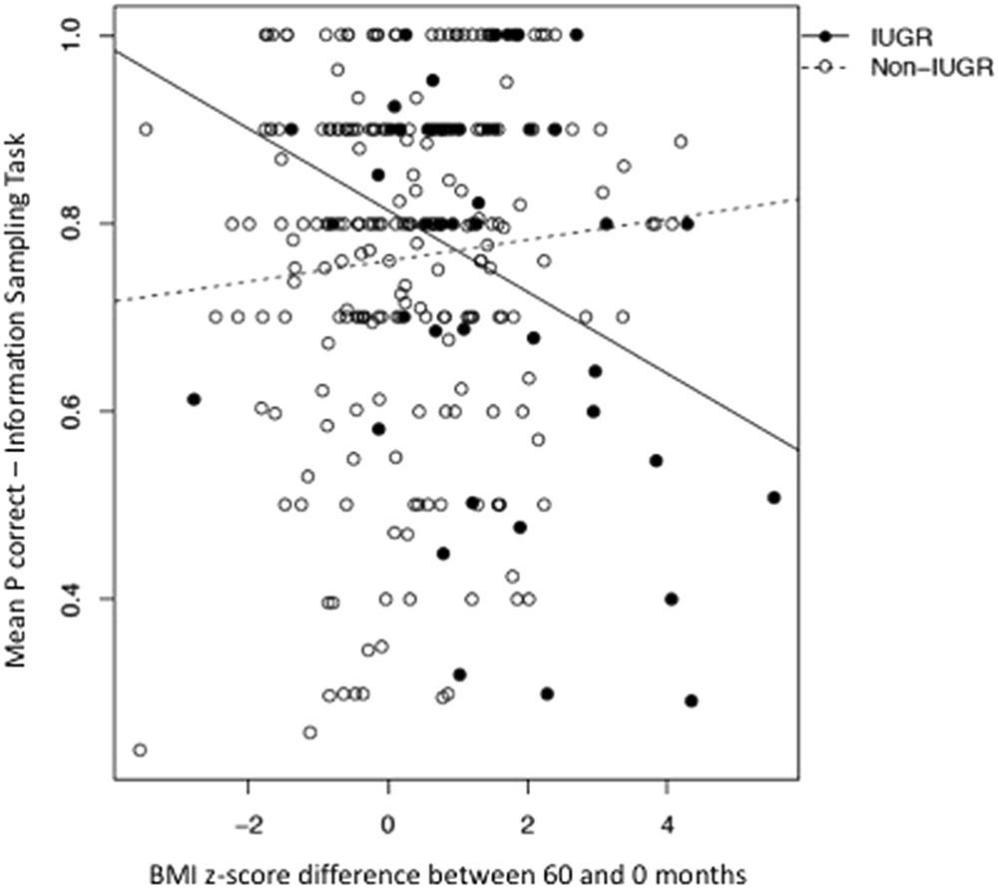
Information Sampling Task (CANTAB at 60 months of age, MAVAN) - mean P correct score, win condition fixed. The lower the score depicted in the y axis, the more impulsive is the child. There is an interaction between being born IUGR (enclosed dots) and variation in BMI z-scores over the first 5 years of life on impulsivity scores, in which greater catch up growth is related to poorer scores (increased reflection impulsivity) in IUGR children.

We found a significant interaction effect between IUGR and catch up growth on IST mean P correct ($\hat{\beta }$ = −0.05, p = 0.01); post hoc analysis showed that while there was no significant association between growth and mean P correct in non-IUGR children ($\hat{\beta }$ =  0.01, p = 0.24), catch up growth was related to lower mean P correct (less certainty when coming up to a decision or higher reflection impulsivity) in IUGR children ($\hat{\beta }$ = −0.04, p = 0.02) (Fig. 2).

There was also a significant interaction effect on discrimination errors ($\hat{\beta }$ = 0.6, p = 0.001); post hoc analysis showed that the relationship between growth and discrimination errors, though not significant, is negative in non-IUGR children ($\hat{\beta }$ = −0.19, p = 0.08). In the IUGR children, catch up growth was associated with higher number of discrimination errors ($\hat{\beta }$ = 0.41, p = 0.04). There were no significant differences between IUGR and non-IUGR children in the IST outcomes (Table 2), and no main effects of catch up growth (Table 3). Table 3 shows the estimated beta coefficients in the analysis of different CANTAB IST outcome measures.

**Table 2:** Means (standard deviations) of Information Sampling Task (IST) measures from the Cambridge Neuropsychological Test Automated Battery (CANTAB) in MAVAN cohort.

IST measures	Non-IUGRs	IUGRs	p-values
**Fixed win condition**			
Discrimination errors	2.33 (2.06)	2.49 (2.27)	0.64
Number of sampling errors	0.33 (0.87)	0.20 (0.50)	0.17
Mean number of boxes opened per trial	21.87 (6.82)	21.41 (6.47)	0.68
Mean P correct	0.76 (0.18)	0.75 (0.20)	0.74

Comparison between IUGR and non-IUGR children on the different outcomes from the IST task (fixed condition). Student’s T test showed no significant differences between the groups.

**Table 3:** Estimated beta coefficients for analyses of different CANTAB IST outcome measures in the fixed win condition in MAVAN cohort.

		Estimated beta coefficients	p-values
Mean P correct	IUGR status	0.05	0.17
	z-score BMI difference 60 to 0	0.01	0.24
	IUGR x BMI difference 60 to 0	−0.05	0.01
Discrimination errors	IUGR status	−0.46	0.29
	z-score BMI difference 60 to 0	−0.19	0.08
	IUGR x BMI difference 60 to 0	0.60	0.001
Presence of sampling errors	IUGR status	0.30	0.57
	z-score BMI difference 60 to 0	0.13	0.35
	IUGR x BMI difference 60 to 0	−0.44	0.17
All boxes opened per trial (yes/no)	IUGR status	−0.25	0.58
	z-score BMI difference 60 to 0	0.07	0.56
	IUGR x BMI difference 60 to 0	−0.11	0.64

Linear regression models were used to investigate the effect of IUGR and catch up growth, as well as their interaction, on the different parameters of the Information Sampling Task (IST) from the Cambridge Neuropsychological Test Automated Battery (CANTAB).

**Figure 3:**
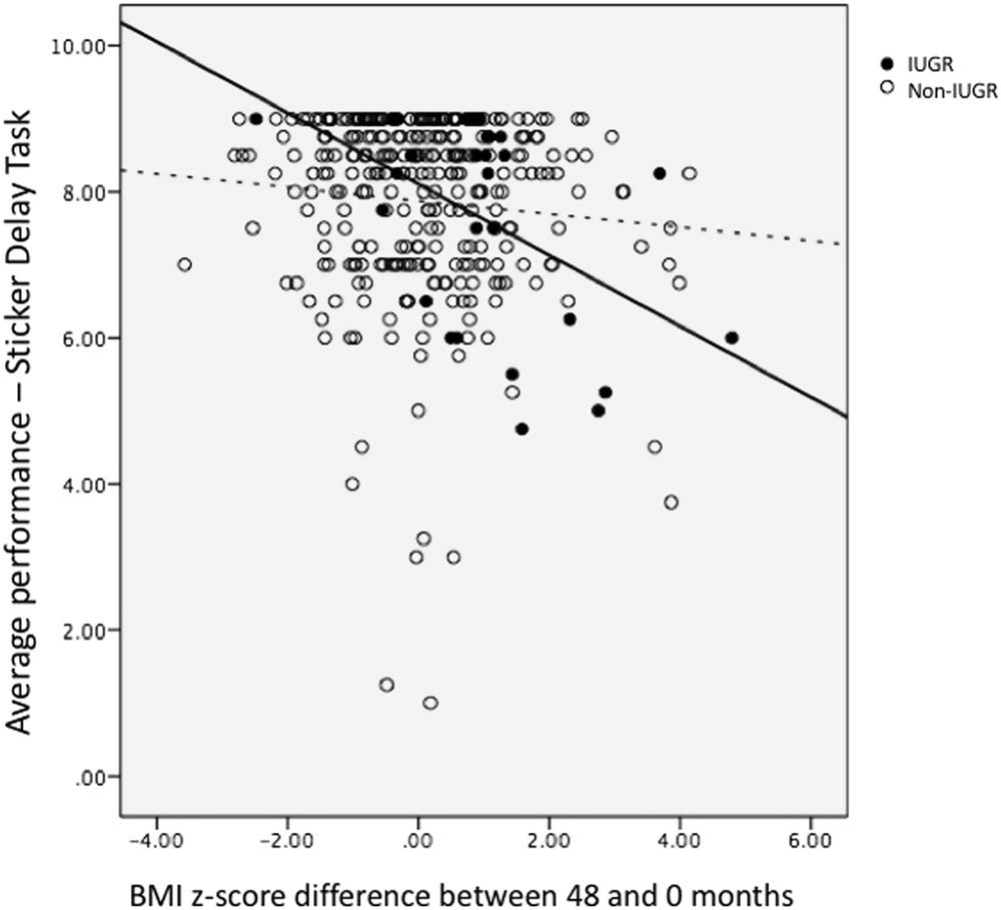
Sticker Delay Task (41 months of age, GUSTO). The lower the score depicted in the y axis, the more impulsive is the child. There is an interaction between being born IUGR (enclosed dots) and variation in BMI z-scores over the first 4 years of life on impulsivity scores, in which greater catch up growth is related to poorer scores (increased impulsivity) in IUGR children.

In the replication cohort, children born IUGR and non-IUGR also did not differ in many confounders as shown in Table 4, except for maternal education. There was a higher percentage of IUGR children whose mothers did not attain University. We saw a significant interaction effect between IUGR and catch up growth on impulsivity levels in GUSTO ($\hat{\beta }$ = 0.39, p = 0.03); post hoc analysis showed that while there was no significant association between growth and impulsivity in non-IUGR children ($\hat{\beta }$ = −0.09 p = 0.11), catch up growth was related to higher impulsivity (lower scores) in IUGR children ($\hat{\beta }$ = −0.49, p = 0.01) (Fig. 3). We repeated the analysis including maternal education in the model, and the results were the same (interaction between IUGR and catch up growth on impulsivity levels, $\hat{\beta }$ = 0.39, p = 0.03; simple slope was significant in the IUGR group $\hat{\beta }$ = −0.50, p = 0.013 but not for the non-IUGR group $\hat{\beta }$ = −0.09, p = 0.11).

**Table 4:** GUSTO participants’ characteristics by birth weight group. Data are expressed as means (standard deviations) or number of participants (percentages).

Sample characteristics	Non-IUGRs (n = 299)	IUGRs (n = 28)	p
Birth weight (kg)^a^	3.20 (0.36)	2.55 (0.17)	<0.0001
Females (%)^b^	158 (52.8%)	15 (53.6%)	1.00
Gestational age (weeks)^a^	38.65 (1.06)	38.86 (1.21)	0.32
Maternal attained education below University (%)^b^	182 (61.7%)	23 (82.1%)	0.04
Family income below $6000 (%)^b^	190 (67.4%)	20 (76.9%)	0.38
Body mass index z-score at 48 months (kg/m^2^)^a^	0.19 (1.19)	−0.28 (1.01)	0.04

^a^Student’s t-test and ^b^Chi-square test.

## Discussion

Here we demonstrated that catch up growth in the first five years of life is related to an objective measure of cognitive/emotional behavior, interacting with fetal growth. We were able to confirm our hypothesis and also previous findings of increased impulsivity in IUGR children^4^, however the tasks used in the current work did not involve food reward. This suggests that IUGR-related impulsivity is not limited to food-associated tasks and may be more general, affecting other forms of this behavior, such as information sampling before making a decision, as well as inhibitory control.

Differently from previous work, the findings reported in this study were not exclusively due to being born IUGR^4^, rather were evident when interacting with growth during childhood. Previously, we have also not seen differences in impulsivity levels towards a sweet reward among IUGR and non-IUGR boys at 36 months of age, only in girls^4^. In young adulthood, however, brain activation in the presence of palatable food images was similarly predicted by birth weight in both boys and girls^6^, as in the current study. It is possible that the pattern of differences in impulsivity changes according to the age of testing, and obviously with the type of task applied, which will be more associated with specific levels of information processing included in the larger construct “impulsivity”^10^, as mentioned in the Introduction.

Interestingly, current and former substance users (either amphetamine or opiates^10^, as well as cannabis^36^) demonstrate reduced information sampling on the IST. Several groups including our own have shown that IUGR individuals spontaneously eat more palatable foods at different ages^7,8,37–39^, and the neurobehavioral mechanisms involved potentially overlap with those related to drug addiction^9^. Poor reflection may represent a potential mechanism for risk-taking behavior, as decision-making may be biased toward salient or immediately rewarding options, and this may be true for both artificial and natural rewards (such as food).

An important finding of this work is a significant effect of interaction between being born IUGR and having rapid catch up growth during infancy on impulsivity levels. Insulin secretion and sensitivity are closely linked to patterns of rapid growth during early life^21^; a recent report described that higher fasting glucose (likely linked to high insulin secretion) predicts poorer performance on tests of inhibitory control^40^. Moreover, baseline insulin secretion relates to offense recidivism among impulsive violent alcoholic individuals^41^, suggesting a fine line between crude measures of glucose metabolism and impulsive behavior. Considering our current findings, an exaggerated rate of growth early in life may not be the best nutritional strategy for IUGR children, not only with regards to metabolic health but also when thinking about emotional and cognitive development, as catch up growth was associated both with increased impulsivity and discrimination errors. Discrimination errors are generally related to poorer attention and/or motivation to perform the task^10^. Some studies suggest that lesions to the orbital-frontal cortex (OFC) are linked to a behavioral profile of impulsivity and risky decision-making, and we have described altered function of the OFC related to IUGR in our rodent studies^42^. However, it is important to consider that here we are studying catch-up growth in BMI, and associations with linear growth can be quite different: studies suggest that faster linear growth (e.g. in height, weight or head circumference) may be associated with improved cognitive outcomes^43–45^, while catch-up growth in BMI can have particularly adverse effects on cognition^46^.

**Figure 4:**
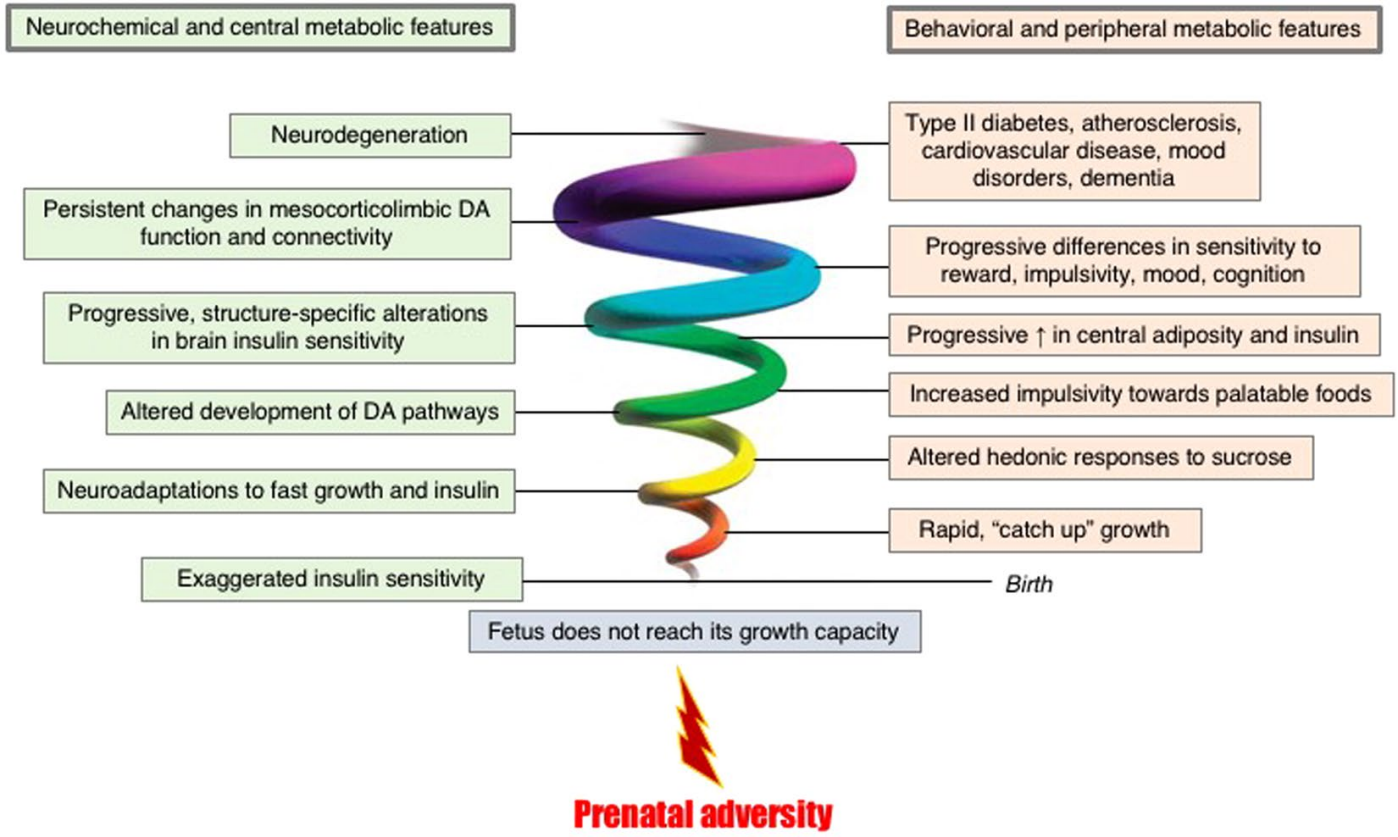
Theoretical framework, life-course upwards spiral (follow the spiral from bottom to top). Please see details in the Discussion section. DA = dopamine.

In Fig. 4, we outline a theoretical framework of the proposed mechanisms involved in these findings and the integration with past findings from our group (follow the spiral from bottom to top). Exposure to prenatal adversity (e.g. placental insufficiency, cigarette smoking, maternal malnutrition, chronic stress) leads to impaired achievement of full growth capacity in utero^47^, with poorer pancreas development^48^. At birth, there is an adaptive exaggerated insulin sensitivity^49^ responsible for a fast, catch up growth^21,49^. However, neuroadaptations occurring in response to the enhanced insulin signaling^50,51^ have a trade-off, represented on the right side of the figure: variability and plasticity of physiological and behavioral responses help the growing organisms to adapt effectively to the uncertainty of later environmental conditions^52^, but at the expense of their health. As a consequence of these neuroadaptations, very specific behavioral features appear already early in life, including altered hedonic responses to sucrose^50,53^. These small, persistent alterations in the behavioral response to sucrose help shaping the development of the dopamine mesocorticolimbic pathways^54^, which in turn triggers altered behaviors such as increased impulsivity^4–6^ and preferences for palatable foods^7,8,37,39,55,56^. All these contribute to a progressive increase in central adiposity and insulin secretion^57,58^, reflected in progressive, region-specific differences in brain insulin function^59^. The initial brain response to a higher availability in circulating insulin is enhanced insulin signaling, which reaches exhaustion at different time-points in different brain regions^60^. In this scenario, the first central region to become insulin-resistant is the hypothalamus^61^. This mosaic of differences in brain insulin function will be detectable in progressive differences in behaviors such as altered sensitivity to reward^62^, impulsivity^63^, mood^64–67^, cognition^40,68,69^, memory^70–72^ and decision-making^41,70^. These modifications in brain insulin function will be accompanied and followed by persistent changes in mesocorticolimbic dopamine function^64,73^ and connectivity between the components of this pathway, such striatal and prefrontal regions^74–77^. The continuation of this process, perpetuated by the behavioral features (e.g. chronic increased intake of high fat high sugar foods), leads to systemic overload and exhaustion, loss of homeostasis, chronic diseases (type II diabetes^1,78^, cardiovascular disease^79^, atherosclerosis^80^, mood disorders^22,81^, Alzheimer’s disease^82,83^) and neurodegeneration^84^. It is interesting to think that genes involved in the regulation of metabolism interact with a variety of environmental layers^85^ and influence the course of these events, likely through diverse epigenetic changes at different ages.

Our study is not free from limitations. Although the replication sample adds strength to the finding, the task used in GUSTO is different from the one used in MAVAN. However, the generalization of these associations to a) an ethnically diverse population and b) other forms of impulsivity such as poor inhibitory control makes our finding potentially relevant. The small sample size of the IUGR group in both cohorts could also explain the lack of main effects from this variable. It is also important to highlight that, although around 90% of all IUGR infants complete their catch up growth by the age of 4 years^86^, some IUGR infants do not exhibit catch-up growth. The underlying causes for their prenatal growth restriction are different, and these causes thus likely lead to a different cascade of events than the one depicted in Fig. 4^87^. Finally, as we did not test food-related impulsivity directly, it is unclear whether the findings represent the identification of a new IUGR/catch-up growth-related deficit or a more-narrow domain that is responsible for previous findings of IUGR-impulsivity deficits, and this needs to be explored in future studies.

## Conclusion

A significant effect of the interaction between fetal growth and postnatal catch up growth on impulsivity levels may be an early signal of vulnerability to both metabolic^1^ and mental health conditions^3^ later in life in IUGR individuals. These findings underscore the importance of awareness and counseling about healthy growth during childhood, especially in those born IUGR.

## Key points

- Being born with low birth weight is related to impulsivity towards food in infancy.
- Here we show that both being born small and growing up fast relate to higher impulsivity in childhood, in two different birth cohorts.
- Impulsivity may be an early signal of vulnerability to both metabolic and mental health conditions later in life in IUGR individuals.
